# Unmet Expectations: miR-34 Plays No Role in p53-Mediated Tumor Suppression In Vivo

**DOI:** 10.1371/journal.pgen.1002859

**Published:** 2012-07-26

**Authors:** Abhinav K Jain, Michelle Craig Barton

**Affiliations:** Program in Genes and Development, Center for Stem Cell and Development Biology, Department of Biochemistry and Molecular Biology, The University of Texas MD Anderson Cancer Center, Houston, Texas, United States of America; Cincinnati Children's Hospital Medical Center, United States of America

In vivo modeling of tumor suppressor p53 functions and regulation has a history of unexpected and even enigmatic outcomes [1], despite the status of p53 as the most frequently mutated gene or dysfunctional pathway in human cancers [2], [3]. Beginning with the surprising viability of the first mice deleted for *Trp53* [4], [5], various hypotheses of compensation, cell type–specificity, stimulus-dependent response, or modifier influences were posed to explain how an exquisitely regulated transcription factor, implicated in a vast array of pathways [6], appeared to have no impact on development. Limited background-specific developmental and fertility problems do occur, especially in female p53-null mice [7], [8], and deletion of potentially compensatory p53 family members, p63 and p73 isoforms, leads to profound developmental and tissue-specific phenotypes [9], [10]. But overall, the most striking result of p53 loss in vivo is early tumor predisposition in p53^−/−^ mice, which lack genomic surveillance provided by p53-mediated regulation of cell cycle arrest, apoptosis, and senescence.

As reported by Concepcion et al. in this issue of *PLoS Genetics* [11], expectations built on cell-based studies of p53 response are again unrealized in mouse models. Previously, multiple in vitro analyses suggested that microRNA (miR)-34 family members are important players in a p53-regulated network of genomic surveillance [12]–[17] (Table 1). Together, these studies strongly supported the view that p53 response to multiple stimuli depended on miR-34, and that ectopic expression of miR-34 was sufficient to elicit p53 response, consistent with miR-34 functioning as a bonafide tumor suppressor. However, Concepcion et al. report that complete inactivation of the entire family of miR-34 genes (miR-34a/b/c) or knockout of each individual miR-34 gene in mice leads to little or no change in p53-mediated functions in tumor suppression [11].

**Table 1:** A list of different in vitro and in vivo model systems used to study miR-34 functions.

Model System	Description	miR-34	Functional Outcome	Ref.
mESCs	Mouse embryonic stem cells	Genetrap-mediated deletion	Decreased spontaneous apoptosis during differentiation	[12]
NSCLCs	Non-small cell lung cancer cells	Overexpression	Inhibits growth	[12]
SW480	p53 mutant colon cancer cells	Overexpression	G1-arrest	[12]
Wi38	Human diploid fibroblasts	Depletion	Protection from Staurosporine-induced apoptosis	[12]
IMR90	Primary lung fibroblasts	Overexpression	Growth inhibition (G1 and G2 arrest), and senescence	[13]
A549	Human alveolar adenocarcinoma cells	Overexpression	G1-arrest	[13]
HCT116	Human colon cancer cells	Overexpression	G1-arrest	[13]
TOV21G	Human ovarian cancer cells	Overexpression	G1-arrest	[13]
MEFs	Mouse embryonic fibroblasts	Overexpression	Apoptosis	[13]
H1299	Human lung cancer cells	Overexpression	Reduced colony formation	[14]
U2OS	Human osteosarcoma cells	Depletion	Reduction in Etoposide-induced apoptosis	[14]
HCT116 (p53^+/+^ or p53^−/−^)	Human colon cancer cells	Long-term overexpression	Apoptosis	[15]
HCT116, RKO	Human colon cancer cells	Overexpression	Suppression of proliferation and induction of senescence	[16]
Mouse xenograft model	HCT116 or RKO cells were inoculated into nude mice	Subcutaneous administration of miR-34a/atelocollagen complexes	Suppression of cell proliferation and reduction in tumor volume	[16]
H1299	Human lung cancer cells	Overexpression	Apoptosis	[17]
U2OS	Human osteosarcoma cells	Overexpression	G1-arrest and reduction in colony formation	[17]
MiaPaCa2, BxPC3	p53 mutant human pancreatic cancer cell lines	Overexpression	Inhibited clonogenic cell growth and invasion, induced apoptosis and G1 and G2 arrest; sensitized the cells to chemotherapy and radiation	[34]
OSN1, OSN2	Neoplastic epithelial ovarian cells	Overexpression	Suppression of proliferation and reduced colony formation	[35]
Kelly, NGP	Neuroblastoma cells with MYCN amplification (+MNA)	Overexpression	Reduction in proliferation and increased apoptosis	[36]
SK-N-AS	Neuroblastoma cells without MYCN amplification (-MNA)	Overexpression	Reduction in proliferation and increased apoptosis	[36]
Mouse mir34a^−/−^; mir34b/c^−/−^	miR-34 knockout mouse in C57BL/6 background	Germline deletion of miR-34	Efficient reprogramming, no effect on proliferation	[30]
Mouse mir34^TKO^	miR-34 knockout mouse in129SvJae and C57BL/6 mixed background	Germline deletion of miR-34	Normal p53 activity	[11]

Interest in a miR-34 axis as mediator of p53-response begins with the niche that miRNAs fill in regulation of RNA expression. miRNAs are small, regulatory non-coding RNAs that generally mediate post-transcriptional silencing of a number of specific target mRNAs [18]. More than 50% of human miRNA genes are found within cancer-associated or fragile sites of the genome, which suggests that miRNAs play essential roles in tumorigenesis [19]. The identification of miRNAs as regulatory targets of p53 [20] suggested their potential involvement in tumor suppression, and expanded the repertoire of p53 downstream targets to both coding and non-coding genes. Further, the view that p53 both positively and negatively regulates gene expression could now rely on increased expression of miRNAs as a mechanism for p53-mediated, indirect repression of gene expression [13], [20], in addition to the few documented cases of direct repression by p53 binding to chromatin [21]–[25].

The members of the evolutionarily conserved miR-34 family, which arise from three different transcripts at two different gene loci in vertebrates, were the first of several non-coding RNAs identified as directly activated by p53 in response to genotoxic stress [13], [26]. miR-34a is at 1p36, a region commonly deleted in tumors, and miR-34b and miR-34c share a common primary transcript arising from 11q23 [27], [28]. miR-34a, b, and c are expressed at very low levels in several types of cancers [28]. Previous reports show that p53 directly activates miR-34a/b/c expression and, dependent on cellular context, they act downstream of p53 in mediating cell cycle arrest or apoptosis [29]. The current list of validated miR-34 downstream targets includes several genes that are repressed during cell cycle arrest or apoptosis when p53 is activated [28].

Given the rationale provided by these studies in cultured cells (Table 1), multiple laboratories created genetic knockout models of either miR-34a or miR-34b/c, or a compound mutant animal harboring homozygous deletion of all three miR-34 family members (miR-34^TKO^) [11], [30]. Surprisingly, mice bearing the miR-34 deletion(s) developed normally, are born at the expected Mendelian ratio, and are fertile [11]. The authors subjected the mice and derived mouse embryonic fibroblasts (MEFs) to a battery of tests to assess any impact on p53-dependent tumor suppression. MEFs obtained from mir-34^TKO^ mice have a slightly higher proliferation rate, but reach senescence with kinetics similar to wild-type MEFs. In response to genotoxic threats, miR-34–deficient MEFs are indistinguishable from wild type: they undergo p53-dependent cell cycle arrest and apoptosis. With ectopic expression of oncogenic K-Ras, p53-deficient MEFs are readily transformed, which is not true of K-Ras–expressing miR-34^−/−^ MEFs.

In the intact mouse, the story is similar: aging cohorts of mir-34^TKO^ mice remain healthy with no spontaneous tumors, in contrast to p53-null mice [4]. In fact, miR-34–deficient mice remain remarkably healthy and tumor-free for at least 60 weeks after irradiation. Assays of apoptosis in response to irradiation proved positive in tissues of these mice, which additionally exhibited no acceleration of tumor progression in Eμ-models of B-cell lymphomagenesis. All of these assessments of p53 functions in vivo undermine the view that miR-34 functions as a tumor suppressor or is an essential component of the p53-tumor suppression network.

Although miR-34 proved nonessential in the most highly studied examples of p53 function (senescence, cell cycle arrest, apoptosis, and tumor suppression), it remains possible that miR-34 is involved in other p53-influenced processes, such as metabolism, autophagy, stem cell quiescence, differentiation, and embryogenesis [6]. For example, specific links between miR-34– and p53-regulated functions have been forged in stem cells [26]. miR-34–deficient MEFs are more efficiently reprogrammed to induced pluripotent stem cells (iPSCs), by expression of pluripotency factors and c-myc [30], compared to wild-type counterparts. While this study of miR-34 as a barrier to reprogramming does not establish a direct tie to p53, it complements multiple reports that depletion of p53 or dysfunctional p53 pathways enhance the efficiency of reprogramming differentiated, somatic cells to iPSCs [31]. Recently, we showed that p53 promotes human embryonic stem cell differentiation by direct activation of p21 and miRNAs, including miR-34a, which repress pluripotency factors and SIRT1 [32]. Taken together, these results indicate that miR-34 has pro-differentiation effects in maintenance of nontransformed, somatic cells, some of which are p53-dependent.

In the future, miR-34–deficient mouse models will be valuable in addressing whether miR-34 functions downstream of p53 in a tissue- and/or context-specific manner. miR-34a, miR-34b, and miR-34c share the same seed sequence and target the same RNAs, although differences in target accessibility or binding affinities may dictate their effectiveness. Genome-wide expression analysis may be needed to determine family member–specific effects, such as the reported regulation of c-MYC by miR-34b/c and not miR-34a [33]. Questions of specificity in gene targets for each member of a miRNA family and potential compensation by other miRNAs may be addressed by studies in these and other miRNA mouse models, perhaps still under development. Non-coding RNAs are thought to act in networks that impact diverse cellular pathways, suggesting considerable challenges ahead in asking the right questions and understanding the functional significance of these RNAs.

## References

[1] Spike BT, Wahl GM (2011) p53, stem cells, and reprogramming: tumor suppression beyond guarding the genome. Genes Cancer 2: 404–419. 21779509 10.1177/1947601911410224 PMC3135646

[2] Soussi T (2007) p53 alterations in human cancer: more questions than answers. Oncogene 26: 2145–2156. 17401423 10.1038/sj.onc.1210280

[3] Hollstein M, Sidransky D, Vogelstein B, Harris CC (1991) p53 mutations in human cancers. Science 253: 49–53. 1905840 10.1126/science.1905840

[4] Harvey M, McArthur MJ, Montgomery CA Jr, Butel JS, Bradley A, et al (1993) Spontaneous and carcinogen-induced tumorigenesis in p53-deficient mice. Nat Genet 5: 225–229. 8275085 10.1038/ng1193-225

[5] Jacks T, Remington L, Williams BO, Schmitt EM, Halachmi S, et al (1994) Tumor spectrum analysis in p53-mutant mice. Curr Biol : CB 4: 1–7. 7922305 10.1016/s0960-9822(00)00002-6

[6] Vousden KH, Prives C (2009) Blinded by the light: the growing complexity of p53. Cell 137: 413–431. 19410540 10.1016/j.cell.2009.04.037

[7] Sah VP, Attardi LD, Mulligan GJ, Williams BO, Bronson RT, et al (1995) A subset of p53-deficient embryos exhibit exencephaly. Nat Genet 10: 175–180. 7663512 10.1038/ng0695-175

[8] Hu W, Feng Z, Teresky AK, Levine AJ (2007) p53 regulates maternal reproduction through LIF. Nature 450: 721–724. 18046411 10.1038/nature05993

[9] Mills AA, Zheng B, Wang XJ, Vogel H, Roop DR, et al (1999) p63 is a p53 homologue required for limb and epidermal morphogenesis. Nature 398: 708–713. 10227293 10.1038/19531

[10] Yang A, Walker N, Bronson R, Kaghad M, Oosterwegel M, et al (2000) p73-deficient mice have neurological, pheromonal and inflammatory defects but lack spontaneous tumours. Nature 404: 99–103. 10716451 10.1038/35003607

[11] Concepcion CP, Han Y-C, Mu P, Bonetti C, Yao E, et al (2012) Intact p53-dependent responses in miR-34-deficient mice. PloS Genet 8: e1002792 doi:10.1371/journal.pgen.1002797. 22844244 10.1371/journal.pgen.1002797 PMC3406012

[12] Bommer GT, Gerin I, Feng Y, Kaczorowski AJ, Kuick R, et al (2007) p53-mediated activation of miRNA34 candidate tumor-suppressor genes. Curr Biol 17: 1298–1307. 17656095 10.1016/j.cub.2007.06.068

[13] He L, He X, Lim LP, de Stanchina E, Xuan Z, et al (2007) A microRNA component of the p53 tumour suppressor network. Nature 447: 1130–1134. 17554337 10.1038/nature05939 PMC4590999

[14] Raver-Shapira N, Marciano E, Meiri E, Spector Y, Rosenfeld N, et al (2007) Transcriptional activation of miR-34a contributes to p53-mediated apoptosis. Mol Cell 26: 731–743. 17540598 10.1016/j.molcel.2007.05.017

[15] Chang TC, Wentzel EA, Kent OA, Ramachandran K, Mullendore M, et al (2007) Transactivation of miR-34a by p53 broadly influences gene expression and promotes apoptosis. Mol Cell 26: 745–752. 17540599 10.1016/j.molcel.2007.05.010 PMC1939978

[16] Tazawa H, Tsuchiya N, Izumiya M, Nakagama H (2007) Tumor-suppressive miR-34a induces senescence-like growth arrest through modulation of the E2F pathway in human colon cancer cells. Proc Natl Acad Sci U S A 104: 15472–15477. 17875987 10.1073/pnas.0707351104 PMC2000550

[17] Tarasov V, Jung P, Verdoodt B, Lodygin D, Epanchintsev A, et al (2007) Differential regulation of microRNAs by p53 revealed by massively parallel sequencing: miR-34a is a p53 target that induces apoptosis and G1-arrest. Cell Cycle 6: 1586–1593. 17554199 10.4161/cc.6.13.4436

[18] Bartel DP (2004) MicroRNAs: genomics, biogenesis, mechanism, and function. Cell 116: 281–297. 14744438 10.1016/s0092-8674(04)00045-5

[19] Calin GA, Sevignani C, Dumitru CD, Hyslop T, Noch E, et al (2004) Human microRNA genes are frequently located at fragile sites and genomic regions involved in cancers. Proc Natl Acad Sci U S A 101: 2999–3004. 14973191 10.1073/pnas.0307323101 PMC365734

[20] He L, He X, Lowe SW, Hannon GJ (2007) microRNAs join the p53 network–another piece in the tumour-suppression puzzle. Nat Rev Cancer 7: 819–822. 17914404 10.1038/nrc2232 PMC4053212

[21] Spurgers KB, Gold DL, Coombes KR, Bohnenstiehl NL, Mullins B, et al (2006) Identification of cell cycle regulatory genes as principal targets of p53-mediated transcriptional repression. J Biol Chem 281: 25134–25142. 16798743 10.1074/jbc.M513901200

[22] Lee KC, Crowe AJ, Barton MC (1999) p53-mediated repression of alpha-fetoprotein gene expression by specific DNA binding. Mol Cell Biol 19: 1279–1288. 9891062 10.1128/mcb.19.2.1279 PMC116057

[23] Lin T, Chao C, Saito S, Mazur SJ, Murphy ME, et al (2005) p53 induces differentiation of mouse embryonic stem cells by suppressing Nanog expression. Nat Cell Biol 7: 165–171. 15619621 10.1038/ncb1211

[24] Riley T, Sontag E, Chen P, Levine A (2008) Transcriptional control of human p53-regulated genes. Nat Rev Mol Cell Bio 9: 402–412. 18431400 10.1038/nrm2395

[25] Ho J, Benchimol S (2003) Transcriptional repression mediated by the p53 tumour suppressor. Cell Death Differ 10: 404–408. 12719716 10.1038/sj.cdd.4401191

[26] Lin CP, Choi YJ, Hicks GG, He L (2012) The emerging functions of the p53-miRNA network in stem cell biology. Cell Cycle 11: 2063–72. 22580472 10.4161/cc.20207 PMC3368858

[27] Versteeg R, Caron H, Cheng NC, van der Drift P, Slater R, et al (1995) 1p36: every subband a suppressor? Eur J Cancer 31A: 538–541. 7576962 10.1016/0959-8049(95)00037-j

[28] Hermeking H (2010) The miR-34 family in cancer and apoptosis. Cell Death Differ 17: 193–199. 19461653 10.1038/cdd.2009.56

[29] Hermeking H (2007) p53 enters the microRNA world. Cancer Cell 12: 414–418. 17996645 10.1016/j.ccr.2007.10.028

[30] Choi YJ, Lin CP, Ho JJ, He X, Okada N, et al (2011) miR-34 miRNAs provide a barrier for somatic cell reprogramming. Nat Cell Biol 13: 1353–1360. 22020437 10.1038/ncb2366 PMC3541684

[31] Krizhanovsky V, Lowe SW (2009) Stem cells: The promises and perils of p53. Nature 460: 1085–1086. 19713919 10.1038/4601085a PMC2974062

[32] Jain AK, Allton K, Iacovino M, Mahen E, Milczarek RJ, et al (2012) p53 regulates cell cycle and microRNAs to promote differentiation of human embryonic stem cells. PLoS Biol 10: e1001268 doi:10.1371/journal.pbio.1001268. 22389628 10.1371/journal.pbio.1001268 PMC3289600

[33] Leucci E, Cocco M, Onnis A, De Falco G, van Cleef P, et al (2008) MYC translocation-negative classical Burkitt lymphoma cases: an alternative pathogenetic mechanism involving miRNA deregulation. J Pathol 216: 440–450. 18802929 10.1002/path.2410

[34] Ji Q, Hao X, Zhang M, Tang W, Yang M, et al (2009) MicroRNA miR-34 inhibits human pancreatic cancer tumor-initiating cells. PloS ONE 4: e6816 doi:10.1371/journal.pone.0006816. 19714243 10.1371/journal.pone.0006816 PMC2729376

[35] Corney DC, Flesken-Nikitin A, Godwin AK, Wang W, Nikitin AY (2007) MicroRNA-34b and MicroRNA-34c are targets of p53 and cooperate in control of cell proliferation and adhesion-independent growth. Cancer Res 67: 8433–8438. 17823410 10.1158/0008-5472.CAN-07-1585

[36] Welch C, Chen Y, Stallings RL (2007) MicroRNA-34a functions as a potential tumor suppressor by inducing apoptosis in neuroblastoma cells. Oncogene 26: 5017–5022. 17297439 10.1038/sj.onc.1210293

